# Analytical Validation and Clinical Application of Rapid Serological Tests for SARS-CoV-2 Suitable for Large-Scale Screening

**DOI:** 10.3390/diagnostics11050869

**Published:** 2021-05-12

**Authors:** Amedeo De Nicolò, Valeria Avataneo, Jessica Cusato, Alice Palermiti, Jacopo Mula, Elisa De Vivo, Miriam Antonucci, Stefano Bonora, Andrea Calcagno, Giovanni Di Perri, Francesco Giuseppe De Rosa, Antonio D’Avolio

**Affiliations:** 1 Department of Medical Sciences, University of Torino, 10126 Torino, Italy; valeria.avataneo@unito.it (V.A.); jessica.cusato@unito.it (J.C.); alice.palermiti@unito.it (A.P.); jacopo.mula@unito.it (J.M.); elisa.devivo59@gmail.com (E.D.V.); miriam.antonucci20@gmail.com (M.A.); stefano.bonora@unito.it (S.B.); andrea.calcagno@unito.it (A.C.); giovanni.diperri@unito.it (G.D.P.); francescogiuseppe.derosa@unito.it (F.G.D.R.); antonio.davolio@unito.it (A.D.); 2 CoQua Lab, 10147 Torino, Italy

**Keywords:** COVID-19, serological test, IgG, IgM, antibodies, immune response, microsampling

## Abstract

Recently, large-scale screening for COVID-19 has presented a major challenge, limiting timely countermeasures. Therefore, the application of suitable rapid serological tests could provide useful information, however, little evidence regarding their robustness is currently available. In this work, we evaluated and compared the analytical performance of a rapid lateral-flow test (LFA) and a fast semiquantitative fluorescent immunoassay (FIA) for anti-nucleocapsid (anti-NC) antibodies, with the reverse transcriptase real-time PCR assay as the reference. In 222 patients, LFA showed poor sensitivity (55.9%) within two weeks from PCR, while later testing was more reliable (sensitivity of 85.7% and specificity of 93.1%). Moreover, in a subset of 100 patients, FIA showed high sensitivity (89.1%) and specificity (94.1%) after two weeks from PCR. The coupled application for the screening of 183 patients showed satisfactory concordance (K = 0.858). In conclusion, rapid serological tests were largely not useful for early diagnosis, but they showed good performance in later stages of infection. These could be useful for back-tracing and/or to identify potentially immune subjects.

## 1. Introduction

To date, the COVID-19 pandemic still represents a major global health problem, with variable severity and mortality across different countries [1].

One of the major challenges associated with the management of this pandemic is reliable epidemiological monitoring. In fact, the rapid spread of SARS-CoV-2 caught many countries off-guard, particularly during the first wave of the pandemic, which revealed a lack of materials or facilities for strict, large-scale biomolecular testing [2,3]. According to current WHO guidelines, reverse transcriptase real-time PCR (rRT-PCR) analysis for the detection of SARS-CoV-2 RNA with nasopharyngeal swabs is considered to be the gold standard for diagnostic purposes [4]. Nevertheless, several works have evidenced some cases of false negatives with PCR testing, particularly when testing upper respiratory tract specimens, mainly due to wrong timing, low viral load, and inter-specimen differences [4,5,6,7,8,9].

Despite these challenges, the application of lockdowns and social distancing, together with general hygienic countermeasures and a progressive improvement in diagnostic capabilities in the vast majority of developed countries, has been quite effective in controlling the pandemic in 2020, significantly reducing the number of deaths [10].

On the other hand, the real prevalence of people who experienced SARS-CoV-2 infection, and who are potentially immune to a new infection, is still difficult to estimate [2,3,8,11,12].

In this scenario, the wide-scale application of serological tests for screening purposes could be beneficial to obtain epidemiological information, which is pivotal for managing the spread of the COVID-19 pandemic in the near future, in addition to the evaluation of the effectiveness of vaccination campaigns [7,8,13,14].

To manage this public health challenge, analytical and clinical validation of serological tests, and a detailed description of the serological characteristics of this disease are crucial tasks; in fact, due to high demand from the market, many serological assays have been introduced, making it difficult to choose a standard [8,15,16,17]. For large-scale and high sensitivity testing, the best serological approach is the evaluation of anti-nucleocapsid (anti-NC) IgGs, which are the most abundant and common during SARS-CoV-2 infection. Moreover, anti-NC IgGs are present only in the case of real symptomatic or asymptomatic infection, but not in the case of vaccination; therefore, their presence can discriminate between a previous or current infection and vaccine response. In addition, anti-N-based assays have been reported to be more sensitive than anti-S-based tests (for the detection of spike protein-specific antibodies), with both IgM and IgG being detected using quantitative RT-PCR as early as 3–4 days after infection and with a peak after 2–3 weeks post-illness onset (or symptom onset) [8,18]. Furthermore, anti-S-based tests displayed cross-reactivity with other coronaviruses, such as Middle East Respiratory Syndrome coronavirus (MERS-CoV), probably due to high conservation of the S2 subunit domain across coronaviruses; cross-reactivity was seen with only the S protein of MERS-CoV and not with the S1 subunit, suggesting more specificity for the S1 subunit-based assays for diagnosing SARS-CoV infections [8,18,19].

Nevertheless, partial information about the correct timing of seroconversion also makes it difficult to interpret the results of these tests. Several works have described the dynamics of immunoglobulin production in SARS-CoV-2-positive patients [11,20,21], highlighting a high proportion (100% in one study [11]) of seroconversion after 2–3 weeks of symptoms. Therefore, we should expect suboptimal analytical performance from serological tests in the first two weeks after symptom onset; nevertheless, there is poor evidence of the prospective performance of these tests compared with rRT-PCR results. Moreover, most of these studies were performed with custom ELISA assays, requiring patients’ serum or plasma samples, and consequently, the drawing of blood [11,20,21].

Considering all the aforementioned issues, in this work we aimed to evaluate the analytical performance and the concordance of two different commercially-available rapid serological tests for anti-SARS-CoV-2 IgG and IgM detection, which are compatible with microsampling and for use on whole blood samples, compared with the rRT-PCR-analysis.

## 2. Materials and Methods

### 2.1. Patient Enrollment

A cross-sectional cohort of patients who underwent PCR testing for SARS-CoV-2 infection with nasopharyngeal swabs, and who were tested for IgG/IgM with a rapid lateral-flow assay (LFA) at several intervals after the biomolecular testing, was enrolled in the context of two ethically approved clinical studies (CORACLE, prot. N. 0000381, 31/03/2020 and e-COVID, N 0065839, 09/07/2020) after giving informed consent. These two protocols aimed, within secondary endpoints, to evaluate the performance of serological testing in a small cohort of hospitalized patients and in exposed healthcare providers. Therefore, little information was available about clinical characteristics and symptoms in the majority of the enrolled subjects.

Other than LFA, an additional serological evaluation was performed in a subset of patients with a semiquantitative bench-top fluorescence immunoassay (FIA).

**Figure 1:**
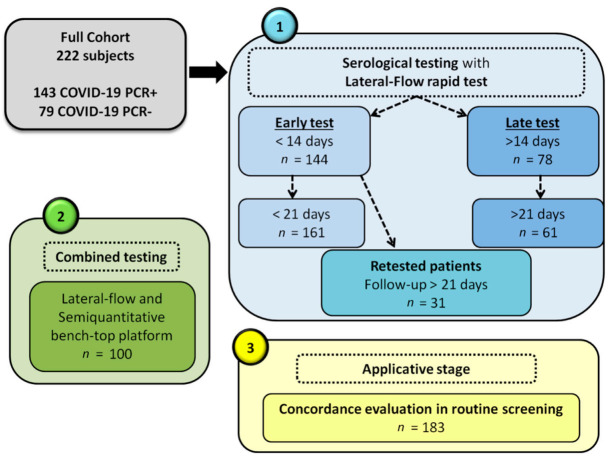
Workflow of patient enrollment and of the evaluation of serological test performance.

After preliminary validation of the LFA and FIA assays in this cohort, the diagnostic concordance of these tests was evaluated in a routine screening setting with coupled samples from 183 patients who had a medium-high risk of COVID-19 infection and with indications for serological screening (particularly healthcare providers or people with a history of COVID-19-compatible symptoms in the previous month, but asymptomatic for at least 2 weeks). A summary of the validation and applicative workflow is depicted in Figure 1.

### 2.2. Confirmation of SARS-CoV-2 Infection

According to WHO recommendations, the diagnosis of SARS-CoV-2 should be based on combined evidence from the clinical history and symptoms, and confirmed with rRT-PCR testing. In our cohort, eventual cases of patients negative on PCR with nasopharyngeal swabs but with a positive contact and symptoms compatible with COVID-19 were retested with PCR. In the case where the PCR testing could not be repeated, confirmation of SARS-CoV-2 infection was based on the combined presence of COVID-19 related symptoms (at least two of the following: fever, myalgia, cough, anosmia, or ageusia), together with a positive contact and combined positive serological tests (positivity on both LFA and FIA after 2 weeks from the onset of symptoms). In order to reduce the bias associated with this issue, this small fraction of cases was considered COVID-19 positive for the validation of the LFA and FIA tests.

### 2.3. Serological Testing

The analytical and diagnostic performance of two different rapid serological tests were evaluated. One is a minimally invasive rapid LFA for anti-nucleocapsid antibodies (COVID-19 IgG/IgM Rapid Test Cassette, RTC; Prima Lab, Balerna, CH, Switzerland), which is compatible with finger pricking and self-reading, is capable of being performed in 10 min and requires 20 µL of blood. This test was performed by the laboratory staff and was interpreted by two independent readers for confirmation. According to the manufacturer’s instructions, the appearance of a faint (e.g., pale-yellow line) or very late response (later than 10–11 min) was considered a negative result.

The second was a semiquantitative FIA for anti-nucleocapsid antibodies (AFIAS COVID-19 Ab test, EOS; Boditech, Chuncheon-si, Korea), performed on 30 µL of blood withdrawn with a capillary tip, which was directly placed in the testing cartridges. Lot-specific calibration was provided by the manufacturer with the reagents and consumables. Semiquantitative results from FIA were expressed in arbitrary units (AU), with a cut-off index for positivity of 1.1 AU and showed saturation beyond 40 AU. For each test, positivity was determined based on at least one positive result either for IgM or IgG. In order to analyze the overall concordance of serology with biomolecular testing, a serological score was calculated by the sum of the binary results (0 vs. 1) of serological tests, and these were compared with the rRT-PCR results.

### 2.4. Statistical Analysis

Statistical analysis was performed with SPSS version 26.0 (IBM, Chicago, IL, USA).

Contingency tables were produced for the evaluation of diagnostic performance and analytical concordance. Diagnostic performance was expressed in terms of sensitivity, specificity, and negative and positive predictive values (NPV and PPV). Then, analytical concordance between serological tests was evaluated with Cohen’s Kappa (k) statistic in order to normalize the results by random concordance. Differences in continuous variables between dichotomic groups were tested with the non-parametric Mann–Whitney test.

## 3. Results

### 3.1. Cross-Sectional Performance of Rapid Serological LFA

A total of 222 patients who received rRT-PCR testing followed by LFA analysis were enrolled.

Among these, 29 were inpatients (excluding intensive care units), nine were negative controls (unexposed individuals who had no symptoms of respiratory illness in 2020), and 13 were potentially exposed healthcare providers who had respiratory symptoms. The other subjects were healthcare providers or hospital personnel who did not disclose their history of COVID-19 symptoms, but who were already tested via rRT-PCR and underwent further LFA testing.

Without considering the amount of time after the PCR testing, the overall performance of LFA was suboptimal both in terms of sensitivity and NPV (sens. 55.9%, spec. 94.9%, NPV 54.3%, PPV 95.2%, k = 0.436). Considering that seroconversion is known to require some time (usually between one and three weeks), statistical analysis was performed according to the delay between the rRT-PCR assay and the LFA testing.

According to this stratification, 144 patients had early LFA testing within 14 days (median 3 days, IQR 0–7); sensitivity and NPV decreased to 40.4% and 46.2%, respectively. Specificity and PPV still remained high (96.0% and 95.0%, respectively, k = 0.291).

When the delay was increased up to 21 days, the analytical performance on 161 patients changed to a sensitivity of 45.6%, a specificity of 94.8%, an NPV of 49.5%, and a PPV of 94.0% (k = 0.337).

By considering the 78 patients who had LFA testing after 14 days with a median of 30 days (IQR 22–42 days), the performance improved to a sensitivity of 85.7%, a specificity of 93.1%, an NPV of 79.4%, and a PPV of 95.5%. Cohen’ s K increased to 0.761, indicating good concordance with biomolecular testing.

Finally, by further selecting only the 61 patients who were tested with LFA after at least 21 days from rRT-PCR (median 37, IQR 27–42), the performance reached a plateau, with a sensitivity of 82.5%, a specificity of 95.2%, an NPV of 74.1%, and a PPV of 97.1% PPV (k = 0.728). All the results have been summarized in Table 1.

### 3.2. Change in Performance during Serological Follow-Up with LFA

In order to further explore the time-dependent diagnostic reliability of LFA, a sub-analysis was performed on a subset of 37 patients who underwent multiple serological tests with LFA (Table 2). These patients were tested within 14 days after rRT-PCR (median 2 days, IQR 0–7 days), and they were re-tested after at least 14 days, with a median of 24 days (IQR 19–27 days).

The performance of LFA increased from a sensitivity of 29.6% to 88.9% and from an NPV of 34.5% to 76.9%, respectively. Specificity and PPV appeared to be perfect (100%) at both timings. The K value changed from 0.185 to 0.812. Reproducing the same evaluation on a subset of 31 patients who were retested after 21 days, the sensitivity was 88.5% and the NPV was 62.5%, comparable to the values achieved after 14 days.

### 3.3. Combined Concordance of Serological Tests with rRT-PCR

Among the 222 patients who were tested with rRT-PCR and LFA, a subset of 100 patients also underwent semiquantitative IgG/IgM determination with an FIA bench-top platform. The results obtained from the two tests were combined into a single score (0: double-negative serological result, 1: only one positive result, 2: double-positive serological result) and compared with the rRT-PCR results, irrespective of clinical status or the timing of PCR testing. Among 40 rRT-PCR-negative patients, 30 (75%) were also negative to both serological tests, three (7.5%) had just one positive test, while seven (17.5%) were double-positive.

Conversely, among 60 rRT-PCR positive patients, 44 (73.3%) were double-positive to serological tests, seven (11.7%) had one positive test, while nine (15.0%) were double-negative to both serological tests (Table 3).

Considering in detail the extremely discordant categories (double-positive serology with negative rRT-PCR results and double-negative serology with positive rRT-PCR results), we observed some peculiar characteristics. Five out of seven patients who appeared as double-positive on serological tests but negative on rRT-PCR testing were likely tested too early (and were not retested due to a shortage in reagents). Since these patients showed COVID-19 symptoms, positive contact, and mutual correlation, they were considered clinically COVID-19 positive.

Among the two remaining patients, one underwent rRT-PCR the day before serological tests, so this patient had likely already cleared the virus. The second one showed only IgM on both serological tests performed 1 week after rRT-PCR testing, which could be explained as a possible early infection, a false negative for biomolecular testing, or a false positive for serology (e.g., cross-reaction). Conversely, double-negative serological results in patients who were positive on rRT-PCR were, as expected, associated with very early serological testing (up to 4 days) in four patients out of nine; the remaining five patients likely did not develop enough adaptive response. Interestingly, two of these patients reported asymptomatic infection.

Therefore, in order to estimate the performance of the combined serological tests excluding the already-identified sources of bias, the analysis was repeated on a subset of 72 patients who were tested after at least 14 days from PCR and considering the patients with clear symptoms, positive contact, and double-positive serological testing as COVID-19 positive. In this case, 15 COVID-19-negative patients (88.2%) were double-negative on serological tests, two (11.8%) were positive on one test (one on the FIA and one to the LFA), and no double-positives were found. Conversely, 44 COVID-19-positive patients (80%) were double-positive on serological tests, six (10.9%) were positive on one test (five to FIA and one to LFA), while five patients (9.1%) were double-negative on serological tests.

### 3.4. Analytical Performance of FIA

As summarized in Table 4, in the group of patients who underwent FIA analysis (*n* = 100), the overall performance in terms of sensitivity, specificity, NPV, and PPV resulted in a sensitivity of 84.6%, a specificity of 88.6%, an NPV of 75.6%, and a PPV of 93.2% (k = 0.704).

Stratifying the 28 patients who were tested before 14 days from rRT-PCR testing, the sensitivity was 60.0%, the specificity was 83.3%, the NPV was 78.9%, and the PPV was 66.7% (k = 0.443). In the 72 patients who were tested beyond 14 days, the performance improved as follows: sensitivity, 89.1%; specificity, 94.1%; NPV, 72.7%; and PPV, 98.0% (k = 0.755).

Repeating the stratification for the tests performed before and after 21 days (36 vs. 64 patients, respectively), the sensitivity changed from 76.5% to 87.5%, specificity from 84.2% to 93.8%, NPV from 80.0% to 71.4%, and PPV from 81.3% to 97.7%, respectively. K changed from 0.609 to 0.736, respectively. Again, performance appeared to reach a plateau after 14 days.

### 3.5. IgG and IgM Detection with FIA

**Figure 2:**
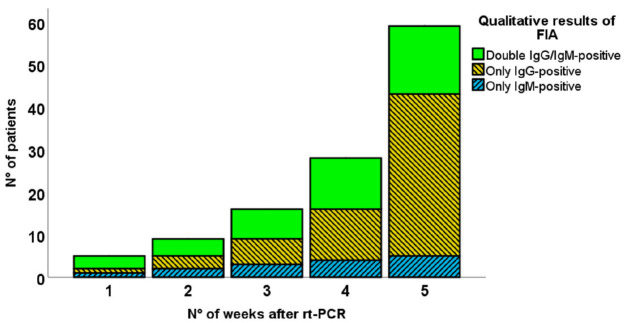
Cumulative results of serological tests relative to the time from first COVID-19 diagnosis through rRT-PCR.

FIA testing allowed a semiquantitative determination of IgG, and IgM was performed. As described in Figure 2, among the 59 positive patients, 38 patients showed only IgG (64.4%), five were positive only to IgM (8.5%), and, finally, 16 were double-positive to IgM and IgG (27.1%). These data demonstrate a higher sensitivity for IgG. According to these results, positivity to IgG and IgM was significantly discordant (Pvalue by McNemar test < 0.001, k = 0.178).

Moreover, the overall sensitivity of IgG for the identification of COVID-19-positive patients was 80.0% (27.7% for IgM), the specificity was 94.3% (91.4 for IgM), the NPV was 71.7% (40.5% for IgM), and the PPV was 96.3% (85.7% for IgM).

The relative proportion of IgM-positive patients decreased over time; in fact, 40% of COVID-19-positive patients who were tested within the first 21 days after biomolecular testing showed IgM positivity, while this proportion fell to 25% in later tested patients. Conversely, the IgG positivity increased over time, ranging from 50% to 85% of COVID-19-positive patients tested within and after 14 days, respectively. In Figure 2, the cumulative frequency of IgM and IgG positivity was resumed.

**Figure 3:**
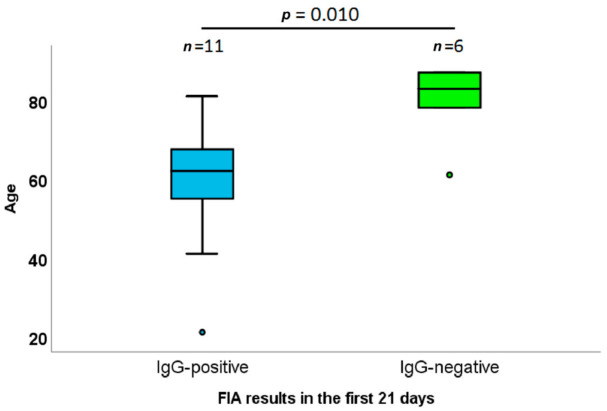
Age distribution among IgG-positive and IgG-negative patients 21 days the first molecular diagnosis of SARS-CoV-2.

Interestingly, among COVID-19-positive patients, those with undetectable IgG during the first 21 days after rRT-PCR positivity (median 11 days, IQR 7–18 days) were significantly older (median 60 vs. 80 years old, Pvalue = 0.010, Figure 3). No other significant difference was evidenced between positive and negative patients. No differences between age classes were observed later than 21 days.

From a quantitative point of view, the median concentrations of IgG and IgM in IgG and IgM-positive patients were 23.1 AU (IQR 16.6–26.4 AU) and 6.8 AU (IQR 3.2–10.1 AU), respectively. No correlation was found between IgG or IgM concentrations with any demographic or anthropometric variable.

### 3.6. Concordance between Serological Tests for Screening Practice

**Figure 4:**
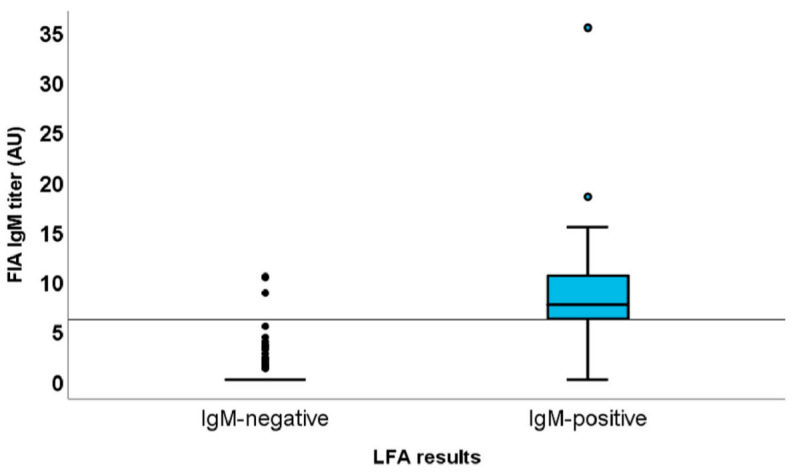
IgM titers lower than six by FIA were associated with IgM false-negative cases with LFA rapid tests.

After the evaluation of the analytical performance of LFA and FIA for the determination of patients’ serological status was completed, the concordance between the two tests was further evaluated by Cohen’s K parameter. The 183 coupled determinations showed high diagnostic concordance, with a mean K value of 0.802 (confidence interval 0.758–0.846). The discordant results mainly concerned IgM positivity due to the low sensitivity of LFA. In fact, as shown in Figure 4, 19 out of 21 samples with an IgM concentration lower than 6 AU with FIA were negative with LFA.

According to the low concordance for IgM positivity, the K value limited to IgG positivity further increased to 0.858 (confidence interval 0.820–0.896), indicating almost perfect concordance.

## 4. Discussion

During 2020, the COVID-19 pandemic strongly affected European countries, with Italy being the first to be hit, particularly the northern regions.

The cumulative number of registered cases in the first wave of the COVID-19 pandemic in Italy (period considered: 24 February to 30 June) was 240,578; however, considering the registered number of deaths (34,767) [22] and the estimated mortality in several studies, the total number of registered cases was obviously underestimated [2]. This has been further confirmed by the analysis conducted by the Italian Institute of Statistics (ISTAT) [23], reporting an average Italian seroprevalence of 2.5%. In fact, by comparing the cumulative number of PCR-confirmed positive cases of COVID-19 at the end of the first wave in the Lombardia region (15 July, *n* = 95,236) with the corresponding seroprevalence (7.5%, *n* ≈ 750,000) the diagnostic rate was as low as 13%. Therefore, “a posteriori” serological screening appears to be the most valuable tool to retrospectively describe the real prevalence of SARS-CoV-2 infection. For this reason, many rapid serological assays have been developed, and as already described in previous works, these can vary significantly in their analytical performance, ranging from ‘completely ineligible’ to ‘almost optimal’ [16,17]. Nevertheless, these studies evaluated the analytical performance of rapid tests performed on serum/plasma samples and did not consider the use of whole blood, which would further increase their applicability but theoretically reduce their sensitivity.

In this work, two serological tests, both based on microsampling (20 and 30 µL of blood, respectively) obtained with finger pricking and fast analysis (10 min), were carried out. An initial result of this work highlights that the reliability of serological testing is strictly time dependent; a delay of at least 14 days between rRT-PCR testing and serological tests is optimal. Concerning IgM and IgG positivity, IgM positivity resulted in lower concentrations and was significantly less present in the observed population, while IgG positivity was prevalent around the second week, reaching the maximum sensitivity before 21 days. A small percentage of patients were negative to serological tests even at 21 days after PCR positivity; this phenomenon could depend on a particularly delayed or absent humoral adaptive immune response [24,25,26,27]. In particular, delayed seroconversion was found to be significantly associated with older age (>80 years old, *p* = 0.010), partially explaining the greater severity of COVID-19 among elderly people. Several reports have showed similar or even higher IgG titers in elderly people [28,29]; therefore, the difference in severity could be related to the timing (more than quantity) of the humoral immune response [24,26]. In fact, these higher IgG titers are probably related to the stronger inflammatory response (particularly Th2 cytokines, e.g., IL-6 [30]) in elderly people [29].

On the other hand, some discordant results between PCR and serology could be explained by false-negative PCR results, thus preventing the serological testing from being 100% concordant [7]. Since these tests appear reliable after 14 days, they can be useful for ‘back-tracing’ purposes in order to suggest (and prioritize) PCR testing for people who have had contact with a serology-positive subject during the previous weeks. Moreover, in light of the current vaccination campaigns [31], which are hindered in several countries (particularly in developing ones) by limited availability, wide-scale serological screening could be a pivotal tool to prioritize the vaccination of seronegative people. The results from this study seem to confirm the WHO indications for the use of rapid serological tests in late stages of the disease (2–3 weeks after the onset of symptoms) to confirm previous SARS-CoV-2 infection [32], while also increasing their applicability by considering the use of microsampling and the direct application of blood samples, in addition to testing their robustness in a careful manner.

Both LFA and FIA are reliable for these purposes, but they have slightly different characteristics. LFA is characterized by a slightly lower and delayed sensitivity, particularly due to low sensitivity for IgM, but it is simpler, cheaper, and immediately useful for ‘point-of-care testing’ (POCT), while FIA retains slightly higher sensitivity, particularly for IgM. Therefore, FIA likely represents a better choice for earlier testing, particularly coupled with PCR, in order to increase the diagnostic sensitivity. The major limit of this study is the lack of clinical information from many participants (e.g., time from the onset of symptoms), as well as its ‘cross-sectional’ nature with considerable variability in the timing of the serological evaluation. A possible selection bias is also present since a more significant proportion of patients were hospitalized in the first months of the pandemic (with severe symptoms), while a higher proportion of asymptomatic healthcare providers and hospital personnel were predominantly enrolled later. Moreover, while the results from this study suggest that the peak of IgG concentration is reached during the second week of infection, in accordance with several other works [11,21,25,33,34], the dynamics of the decline in IgG concentration are still poorly described and could show considerable inter-patient variability. Some recent studies showed IgG persistence for at least 50 days, but little information is available for a longer follow-up period [35,36]. Moreover, one of these studies also showed that a small fraction of patients recovered from COVID-19 without a detectable IgG response [35], whereas others can lose IgG titer while still remaining immune [12]. This evidence, other than suggesting the involvement of an effective innate or adaptive cell-mediated immune response against SARS-CoV-2, indicates that a fraction of cases cannot be diagnosed by serological tests and, therefore, this prevents their sensitivity from reaching 100%. This hypothesis was recently confirmed in a study by Sekine et al. [27], which highlighted that a significant proportion of exposed individuals, patients with very mild or asymptomatic disease, clear the infection through a cell-mediated response without developing detectable Ig titers.

All these issues will be the main topics of future studies.

## 5. Conclusions

Other than confirming the WHO indications for the use of rapid serological tests in late stages of the disease, this work highlights the suitability of the investigated LFA and FIA rapid tests for their application in wide-scale screening. They proved to be reliable and mutually concordant, they have the important advantage of being compatible with microsampling and can be performed directly on blood samples (in contrast to normal serological tests). The sensitivity of these tests was in accordance with that reported in several works, with a delayed seroconversion (2–3 weeks after the onset of symptoms) confirming the poor reliability of these assays for the early detection of SARS-CoV infection. Furthermore, our study suggests particular caution for their use in elderly people, since delayed seroconversion was observed (>21 days). Our findings confirm the potential of rapid serological tests for back-tracing purposes in addition to potentially guiding vaccination campaigns and prioritizing their administration to seronegative people in cases of vaccine shortage.

## Figures and Tables

**Table 1:** Analytical performance of LFA, compared with the ‘gold standard’ biomolecular testing as a reference. The performance has been evaluated relative to the time of testing.

OVERALL	LFA Result			Sensitivity	55.9%
rRT-PCR result	negative	positive	total	Specificity	94.9%
negative	75	4	79	NPV	54.3%
positive	63	80	143	PPV	95.2%
total	138	84	222	Cohen-K	0.436
≤14 DAYS	LFA Result			Sensitivity	40.4%
rRT-PCR result	negative	positive	total	Specificity	96.0%
negative	48	2	50	NPV	46.2%
positive	56	38	94	PPV	95.0%
total	104	40	144	Cohen-K	0.291
>14 DAYS	LFA Result			Sensitivity	85.7%
rRT-PCR result	negative	positive	total	Specificity	93.1%
negative	27	2	29	NPV	79.4%
positive	7	42	49	PPV	95.5%
total	34	44	78	Cohen-K	0.761
≤21 DAYS	LFA Result			Sensitivity	45.6%
rRT-PCR result	negative	positive	total	Specificity	94.8%
negative	55	3	58	NPV	49.5%
positive	56	47	103	PPV	94.0%
total	111	50	161	Cohen-K	0.337
>21 DAYS	LFA Result			Sensitivity	82.5%
rRT-PCR result	negative	positive	total	Specificity	95.2%
negative	20	1	21	NPV	74.1%
positive	7	33	40	PPV	97.1%
total	27	34	61	Cohen-K	0.728

rRT-PCR = reverse transcriptase real-time PCR; LFA = Lateral Flow Assay; NPV = Negative Predictive Value; PPV = Positive Predictive Value.

**Table 2:** Evaluation of the change in analytical performance of LFA in patients who underwent multiple tests. The timing is relative to the rRT-PCR testing.

≤14 DAYS	LFA Result			Sensitivity	29.6%
rRT-PCR result	negative	positive	total	Specificity	100%
negative	10	0	10	NPV	34.5%
positive	19	8	27	PPV	100%
total	29	8	37	Cohen-K	0.185
>14 DAYS	LFA Result			Sensitivity	88.9%
rRT-PCR result	negative	positive	total	Specificity	100%
negative	10	0	10	NPV	76.9%
positive	3	24	27	PPV	100%
total	13	24	37	Cohen-K	0.812
≤21 DAYS	LFA Result			Sensitivity	23.1%
rRT-PCR result	negative	positive	total	Specificity	100%
negative	5	0	5	NPV	20%
positive	20	6	26	PPV	100%
total	25	6	31	Cohen-K	0.088
>21 DAYS	LFA Result			Sensitivity	88.5%
rRT-PCR result	negative	positive	total	Specificity	100%
negative	5	0	5	NPV	62.5%
positive	3	23	26	PPV	100%
total	8	23	31	Cohen-K	0.712

rRT-PCR = reverse transcriptase real-time PCR; LFA = Lateral Flow Assay; NPV = Negative Predictive Value; PPV = Positive Predictive Value.

**Table 3:** Combined concordance of LFA and FIA tests with rRT-PCR. Serological scores indicate the number of positive tests (0 = both negative, 1 = one positive, 2 = both positive). ‘COVID-19 confirmed diagnosis’ includes as positive, in addition to the PCR-positive patients, five patients who had negative PCR testing before the onset of symptoms, but who had clear COVID-19 symptoms. They were mutually correlated, with confirmed positive contact and double-positive serological testing.

OVERALL	Serological Score						
rRT-PCR result	0 (N of patients)	%	1 (N of patients)	%	2 (N of patients)	%	total
negative	30	75.0	3	7.5	7	17.5	40
positive	9	15.0	7	11.7	44	73.3	60
total	39		10		51		100
≤14 Days and COVID-19 Confirmed Diagnosis	Serological Score						
rRT-PCR result	0 (N of patients)	%	1 (N of patients)	%	2 (N of patients)	%	total
negative	15	83.3	1	5.6	2	11.1	18
positive	4	40.0	1	10.0	5	50.0	10
total	19		2		7		28
>14 Days and COVID-19 Confirmed Diagnosis	Serological Score						
rRT-PCR result	0 (N of patients)	%	1 (N of patients)	%	2 (N of patients)	%	total
negative	15	88.2	2	11.8	0	0.0	17
positive	5	9.1	6	10.9	44	80.0	55
total	20		8		44		72

rRT-PCR = reverse transcriptase real-time PCR.

**Table 4:** Analytical performance of FIA compared with the gold standard biomolecular (and clinical) diagnosis of COVID-19.

OVERALL	FIA Result			Sensitivity	84.6
rRT-PCR result	negative	positive	total	Specificity	88.6
negative	31	4	35	NPV	75.6
positive	10	55	65	PPV	93.2
total	41	59	100	Cohen-K	0.704
≤14 DAYS	FIA Result			Sensitivity	60.0
rRT-PCR result	negative	positive	total	Specificity	83.3
negative	15	3	18	NPV	78.9
positive	4	6	10	PPV	66.7
total	19	9	28	Cohen-K	0.443
>14 DAYS	FIA Result			Sensitivity	89.1
rRT-PCR result	negative	positive	total	Specificity	94.1
negative	16	1	17	NPV	72.7
positive	6	49	55	PPV	98.0
total	22	50	72	Cohen-K	0.755
≤21 DAYS	FIA Result			Sensitivity	76.5
rRT-PCR result	negative	positive	total	Specificity	84.2
negative	16	3	19	NPV	80.0
positive	4	13	17	PPV	81.3
total	20	16	36	Cohen-K	0.609
>21 DAYS	FIA Result			Sensitivity	87.5
rRT-PCR result	negative	positive	total	Specificity	93.8
negative	15	1	16	NPV	71.4
positive	6	42	48	PPV	97.7
total	21	43	64	Cohen-K	0.736

rRT-PCR = reverse transcriptase real-time PCR; FIA = Fluorescent ImmunoAssay; NPV = Negative Predictive Value; PPV = Positive Predictive Value.

## Data Availability

Data will be provided upon request.
